# Gratuitous Medical Advice

**Published:** 1882-04

**Authors:** 


### GRATUITOUS MEDICAL ADVICE.

If a hundred individuals, each afflicted with a different ailment, were to come successively into the company of a dozen persons, there is not one of them who would not be told how he could get cured, with the corroborating fact, that the same means had been effectual in the case of "*Mr. Smith, who was a great deal worse off*" than the unfortunate listener, who, if he *tries it*, will only have to add his experience to that of countless thousands who have gone before him, that however efficient it may have proved for others, as for him, it utterly failed of any real good. Or of any hundred newspapers taken up promiscuously, either one of the hundred unfortunates would soon light upon the advertisement of a remedy which suited his case precisely, with the special or implied endorsement of the editor to the same effect, and yet, how has hope been deferred, and the expectant heart sickened and perished in its waiting, all of us understand; and yet, what Mrs. Grundy says, and "I saw it in a newspaper," is gospel to the masses, and to the ignorant amongst the oligarchy of whom there are not a few.

### CHILDREN EATING.

When a child is observed to have little or no appetite for breakfast, sickness of some kind is impending. If in addition to this indifference for food in the morning, there is a uniform desire for a hearty supper at the close of the day, a dyspepsia for life will be founded, which will embitter many an otherwise happy hour; or some other form of chronic disease will result, which medical skill for many years, will often fail to eradicate.

This want of appetite in the morning, and this over appetite late in the day, is the creator of disease in multitudes of grown persons who have reached maturity in good health, but whose change of position, of business, or of associations, has gradually led to the perversion of nature's laws.

Young children naturally, in common with the animal creation, are greedy for breakfast, after the long abstinence of twelve hours, this is the natural arrangement, and it is wise.

Those who do not work hard, and who will have supper, "Tea," should, especially where there are children, have nothing more inviting than cold bread and butter; "Relishes," such as cake or chipped beef and the like, however "simple" they may be, are only stimulants to eating, when nature does not require any thing, and we goad her at our peril.

